# Sensorization of microfluidic brain-on-a-chip devices: Towards a new generation of integrated drug screening systems

**DOI:** 10.1016/j.trac.2023.117319

**Published:** 2023-11

**Authors:** Attilio Marino, Matteo Battaglini, Marie Celine Lefevre, Maria Cristina Ceccarelli, Kamil Ziaja, Gianni Ciofani

**Affiliations:** a Istituto Italiano di Tecnologia, Smart Bio-Interfaces, Viale Rinaldo Piaggio 34, 56025, Pontedera, Italy; b Scuola Superiore Sant’Anna, The Biorobotics Institute, Viale Rinaldo Piaggio 34, 56025, Pontedera, Italy; c University of Aveiro, Department of Chemistry, CICECO-Aveiro Institute of Materials, Rua de Calouste Gulbenkian 1, 3810-074, Aveiro, Portugal

**Keywords:** Brain organoids, Brain-on-a-chip, Blood-brain barrier, Vascularized brain models, Optical sensors, Electrical sensors, Electrochemical sensors, Genetically-encoded sensors, Digital immunosensors

## Abstract

Brain-on-a-chip (BoC) devices show typical characteristics of brain complexity, including the presence of different cell types, separation in different compartments, tissue-like three-dimensionality, and inclusion of the extracellular matrix components. Moreover, the incorporation of a vascular system mimicking the blood-brain barrier (BBB) makes BoC particularly attractive, since they can be exploited to test the brain delivery of different drugs and nanoformulations. In this review, we introduce the main innovations in BoC and BBB-on-a-chip models, especially focusing sensorization: electrical, electrochemical, and optical biosensors permit the real-time monitoring of different biological phenomena and markers, such as the release of growth factors, the expression of specific receptors/biomarkers, the activation of immune cells, cell viability, cell-cell interactions, and BBB crossing of drugs and nanoparticles. The recent improvements in signal amplification, miniaturization, and multiplication of the sensors are discussed in an effort to highlight their benefits *versus* limitations and delineate future challenges in this field.

## 1 Introduction

**Fig. 1:**
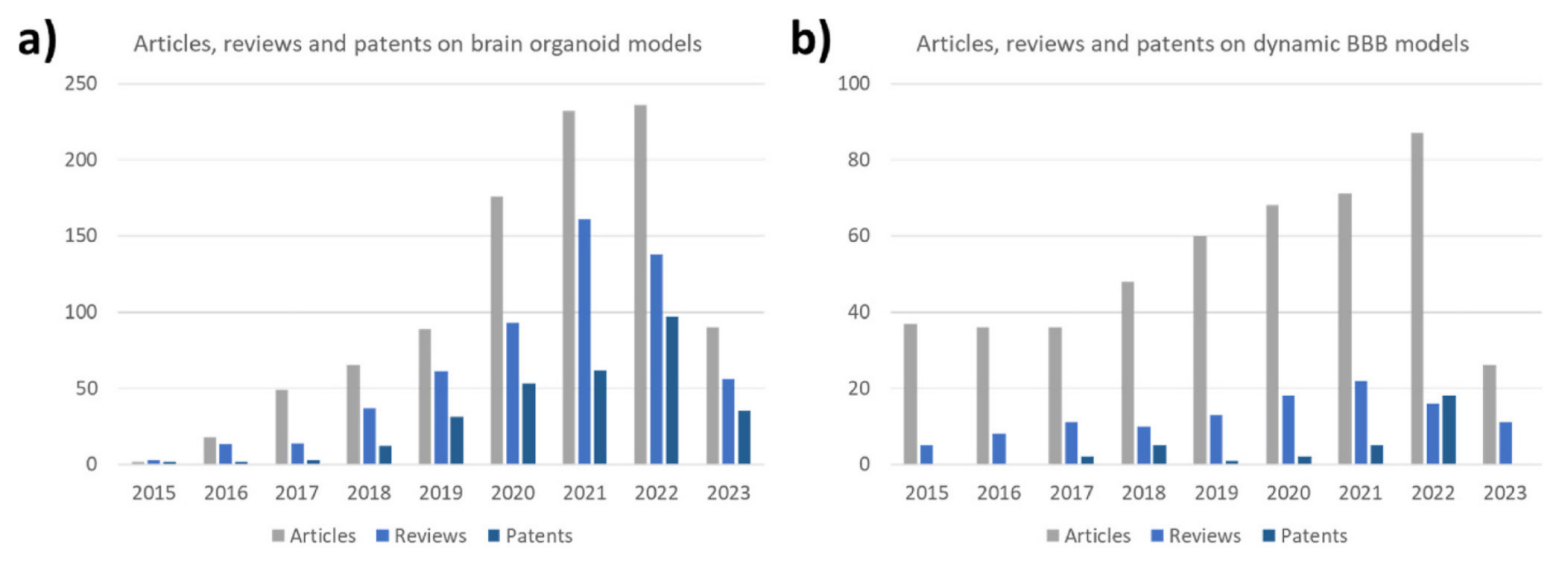
The growing number of research articles, reviews, and patents on a) brain organoid and b) microfluidic dynamic BBB models from 2015 to 2023 (data up to May 31st, 2023).

Biomimetic brain-on-a-chip (BoC) devices are brain-mimicking platforms exploited by public research centers, contract research organizations, and pharmaceutical companies to model specific physio/pathologic conditions and to perform *in vitro* pre-clinical drug screening. BoC systems have been used to study and perform drug testing on pathologic models including neurodegenerative disorders (*e.g*., Parkinson’s, Huntington’s, and Alzheimer’s diseases), ischemia, and neuroinflammation [1]. Moreover, they have been exploited to model and investigate physiologic phenomena of the central nervous system (CNS), such as neural network development (*e.g*., vascularization, neurite outgrowth, and synaptogenesis), cell-cell communication, and immune function [1]. The interest in such brain-mimicking models has remarkably grown in recent years (Fig. 1a shows the growing number of articles, reviews, and patents on brain organoid models from 2015). Considering that FDA no longer has to require to carry out animal tests before clinical trials [2], the use of advanced *in vitro* models is expected to further grow in the next years.

Brain-mimicking *in vitro* models differentiate from standard *in vitro* culture systems since they show typical features of tissue complexity, such as multicellularity, three-dimensionality, vascularization, and the presence of extracellular matrix surrounding cells. Such 3D brain-mimicking models demonstrate superior prediction capabilities compared to simple 2D monolayer cultures [3–6]. Furthermore, their complexity level permits the evaluation of multiple correlated phenomena in a single study, such as drug delivery from the vasculature, drug targeting, drug efficacy in targeted cells, and side effects on healthy cells [7]. Nevertheless, they show remarkably higher accessibility compared to the animal brain, therefore facilitating the control of the experimental conditions as well as the monitoring of the treatment effects. However, the use of BoC devices for carrying out high-throughput investigations and real-time monitoring of experimental results is still challenging. To these aims, the emerging trends involve the incorporation of sensors [8] and the miniaturization of the models [9]. The exploitation of scalable microfabrication technologies is instead required for industrialization.

A fundamental feature of the BoC model is the prediction capability, that is the ability of the system to predict specific phenomena as well as the outcomes of a treatment. Highly predictive BoC are extremely valuable in the selection of the most promising CNS drug candidates, limiting ethical concerns on animal studies and with a remarkable socio-economic impact. As introduced above, the prediction capability is strictly connected to the model biomimicry. In this regard, the incorporation of patient-derived human cells (such as primary cells and induced pluripotent stem cells - iPSCs -) is a recent trend that allows not only to improve the model accuracy, yet also to test patient-tailored target therapies and other personalized treatments [10,11]. As another example, the use of tissue-specific and disease-associated extracellular matrix components to develop brain-mimicking models is gaining interest in the scientific community [12]. This approach particularly suits brain cancer investigations, to examine the mechanisms of cancer cell migration/invasion as well as to test drugs acting on those biological pathways [13].

A particularly important characteristic to replicate into BoC devices is the brain microvasculature, especially for drug delivery applications. The brain is a highly vascularized organ and the blood-brain barrier (BBB) protects it from harmful compounds, viruses, bacteria, and particles. The BBB strongly limits the crossing of pharmaceutical drugs for CNS treatment. Not adequate BBB crossing ability represents a basic criterion for the selection of promising drug candidates, the failure of which results in the termination of drug testing [14]. For this reason, BoC models incorporating a vascularization system are particularly attractive for drug screening purposes (Fig. 1b shows the growing number of articles, reviews, and patents on microfluidic dynamic BBB models from 2015). Such BBB-on-a-chip models can be used to analyze the dynamics of drug/nanomedicine crossing, to support the drug development phases (*e.g*., in the selection of the drug analogs most efficient in BBB crossing), and to monitor the efficacy and safety of BBB-opening technologies (*e.g*., microbubbles) [15].

In this review, we discuss recent trends in BoC devices and their implementation with innovative electric, electrochemical, and optical sensors for real-time monitoring and high-throughput testing, highlighting microfluidic platforms for BBB testing capabilities.

## 2 Brain delivery investigations: from static to microfluidic BBB systems

BBB is a continuous structure enveloping brain blood vessels and separating the bloodstream from the brain environment. The main function of the BBB is to regulate the passage of ions, molecules, and cells from the bloodstream to the CNS and block the passage of potentially harmful substances [16]. The BBB is a part of the so-called neurovascular unit (NVU) which is composed of various cellular components such as brain endothelial cells, pericytes, astrocytes, neurons, microglia, and extracellular components like basement membrane or extracellular matrix [17]. Despite its physiological functions, the BBB represents one of the main obstacles in the treatment of CNS disorders being able to block the passage of most commonly used drugs and therapeutical molecules [18]. Moreover, BBB dysfunctions have been identified and connected to most CNS disorders, including brain cancers and neurodegenerative diseases [19]. The study of BBB functions both in healthy and pathological conditions and the assessment of BBB crossing abilities of candidate drugs thus represent a pivotal topic for the development of treatments for CNS disorders [20]. Currently, *in vivo* testing represents the most widely used approach to model BBB and assess the ability of candidate drugs to reach the CNS. However, the use of animals for *in vivo* testing is time-consuming and expensive, presents a relatively low reproducibility and low translatability of the obtained results in humans, and is affected by the ethical concerns connected to the use of living animals [20]. Therefore, there is a strong need for *in vitro* models able to replicate the *in vivo* conditions of the NVU.

The most common *in vitro* models of the BBB are the Transwell inserts, which are 2D porous membranes of various sizes and materials [21,22]. These inserts are usually seeded with brain endothelial cells on top of the porous membrane and then placed in a multi-well cell culture plate. The obtained *in vitro* BBB model is therefore composed of an apical (luminal) and a basolateral (abluminal) compartment separated by a layer of brain endothelial cells (other components of the NVU such as astrocytes of pericytes can also be introduced) [21,22]. To assess the integrity of the obtained BBB *in vitro* models, various tests can be carried out: the commonly used tests are transendothelial electrical resistance (TEER) measurements [23], permeability tests involving the use of fluorescent tracers [24], and protein expression analysis [25]. TEER analysis refers to the measurement of the electrical resistance posed by a brain endothelial cell layer, and is a quantitative measurement of the barrier coverage and tight junctions’ integrity. TEER measurement is commonly carried out by positioning two electrodes, one in the luminal and one in the abluminal side of the model, and by applying alternating current [23,26]. The TEER is then calculated by applying Ohm’s law as a function of the voltage and current applied [23,26]. Permeability tests are commonly carried out by administrating a fluorescent tracer *(e.g*., fluorescent dextrans of various molecular weights) to the luminal side of the model, and by monitoring its passage across the BBB cell layer over time [24,26]. Lastly, the analysis of protein expression on BBB *in vitro* models is usually carried out by immunostaining to assess the presence of cellular localization of specific markers of tight junctions like claudins (CLDN), zonula occludens-1 (ZO-1), and occludin [OCLN) [25,26].

Currently, the most used *in vitro* BBB systems rely on commercial Transwell inserts, that consist of simple permeable supports placed in multi-well cell culture plates. Inserts are available in different materials, sizes, and pore diameters, thus representing very versatile and cheap platforms for *in vitro* BBB crossing assays. Nevertheless, these models present several limitations: first, they only allow 2D cell cultures, failing at recapitulating perivascular 3D cell arrangement. Second, they underestimate the importance of physiological shear stress on endothelial cells, needed for the achievement of stringent barriers (by the development of tight junctions). These drawbacks result in models that poorly mimic *in vivo* barriers, with consequent unreliable outcomes [26]. This all considered, the interest in the production of new microfluidic 3D BBB *in vitro* models, with a higher degree of biomimicry, literally exploded in the latest years. Recent literature identifies two main BBB model classes: “microfluidic 3D vasculogenesis models” and “microfluidic 3D perivascular devices” [27]. The former exploits the spontaneous tubulogenic properties of endothelial cells into hydrogels to form capillary networks; despite the high biomimetic complexity of micro-vessel networks that can be attained, this model is still far from actual applications in high-throughput screenings, due to difficult shear stress control and sampling in each vessel. The latter presents a design defined by the operator to reproduce the *in vivo* BBB complex architecture and function [28]. The main research effort in the modeling of BBB *in vitro* is placed on the improvement of the biomimetic properties of microfluidic devices, and in the next section, we will analyze the most recent development in this direction.

## 3 Innovative microfluidic brain-on-a-chip models

BoC models combine cell culture, microfabrication, and microfluidics technology to mimic structures and functions of the brain, and to study the complex interactions between the brain environment and the NVU. One of the main research trends related to *in vitro* BoC models concerns the geometry of these devices and the spatial organization of the cellular components used to replicate the NVU and other biological structures.

These microfluidic devices can be designed by using different strategies based on the spatial arrangement of the “blood” and “brain” compartments. The double-layer configuration is an improved version of the Transwell model which includes an upper and a lower chamber separated by a porous membrane. While most models using this configuration are seeded with cells forming 2D layers, recently there has been a shift towards platforms exploiting 3D matrixes to mimic the *in vivo* physiological distribution of the NVU cells. In this regard, Ahn et al. fabricated a microfluidic device in PDMS consisting of two chambers separated by a porous membrane with 8 μm-diameter pores where the physiology of the BBB is mimicked through a 2D endothelial monolayer in the upper compartment, pericytes accommodation underneath the membrane, and astrocytes in 3D Matrigel located in the lower layer [29]. This model was used to assess the passage of HDL-mimetic nanoparticles engineered with apolipoprotein A1 (eHNP-A1), demonstrating the potential of this nanoplatform in crossing the BBB through a transcytosis-mediated mechanism [29].

Despite this vertical spatial arrangement of the microfluidic chip appears to mimic the main characteristics of the BBB, it poses a limitation on simultaneous visualization of all the involved cell types, thereby hindering microscopic analysis. To overcome these limitations, the adoption of a planar side-by-side configuration may be considered. This configuration is composed of two or more parallel horizontal microfluidic channels separated by an array of micropillars. The BBB-on chip proposed by Lee et al. is a PDMS microfluidic system composed of four channels compartmentalized by arrays of micro-pillars having a triculture system with functional barrier properties. They observed how human brain microvascular endothelial cells (HBMECs) and human brain vascular pericytes (HBVPs) sprout and protrude to the opposite side of the channel containing human astrocytes (HAs) in fibrin hydrogel, thus developing a 3D blood vessel network [30].

**Fig. 2:**
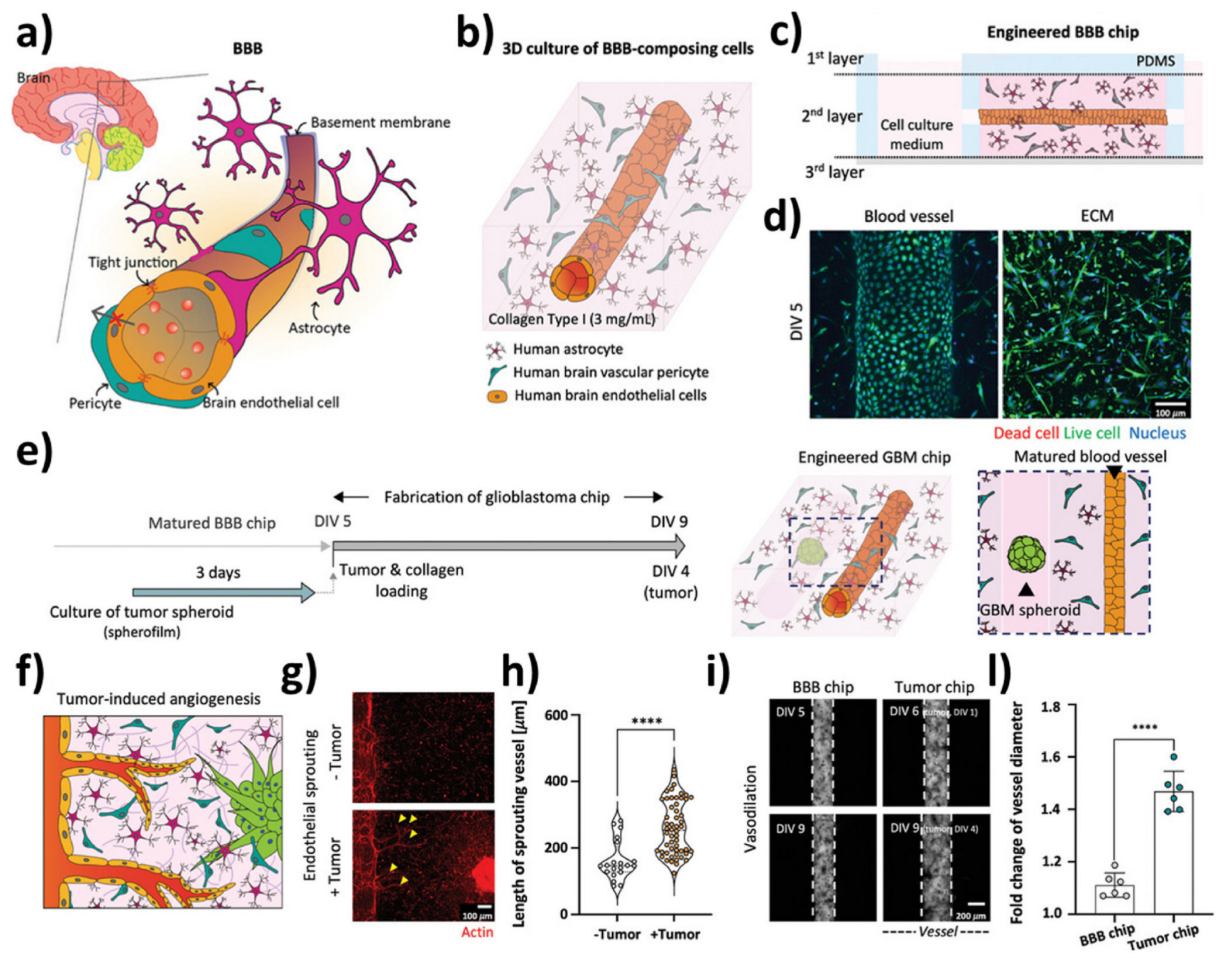
Biomimetic microfluidic BoC mimicking the NVU-tumor interactions in a collagen-based extracellular matrix. Schematic representations of a) the *in vivo* NVU, b) the *in vitro* fluidic NVU model with human brain endothelial cells, human brain pericytes, and human astrocytes (BBB chip), and c) the cross-section of the microfluidic system. d) Imaging of the cells developing the blood vessel (left) and of the cells in the extracellular matrix (ECM). e) BBB chip incorporating a 3D GBM (tumor chip): f) schematic representation, g) imaging, and h) analysis of the tumor-induced angiogenesis. i) Imaging and l) analysis of the NVU dilation induced by the presence of the tumor. Adapted with permission from Ref. [31].

The above-mentioned configurations are suitable for verifying the maturity and the integrity of the BBB through permeability assays, expression of tight junction proteins, and TEER measurement. However, they have limitations related to modeling shear stress on the endothelial component of the barrier. Due to the fabrication process, the double-layer and side-by-side configurations have a prismatic geometry that makes it challenging to attain uniform shear stress in the models: upgraded 3D configurations with a tubular design featuring cylindrical channels have been developed to address this issue, such as the triculture tubular BBB model proposed by Seo et al. In their work, Seo and colleagues realized a co-culture with perivascular cells in a 3D hydrogel matrix in a PDMS device using micro-needles. After gelation, the micro-needles are extracted and the endothelial cells are seeded into the hollow collagen cylinder, resulting in the formation of a cylindrical brain endothelium surrounded by pericytes and astrocytes [31] (Fig. 2). This model not only is a valuable representation of the physiological BBB, yet it has also been utilized for investigating the effect of glioblastoma multiforme (GBM) on the NVU. The interaction between GBM spheroids and the matured BBB induced an increase in length and number of new vessels, and a dilatation of the blood vessels resulting in increased permeability, thus providing a platform for drug screening in pathological conditions. In 2019, our group proposed a BoC microfluidic platform to assess both the BBB crossing efficiency and the anticancer properties of nultin-3a and superparamagnetic iron oxide nanoparticles-loaded solid lipid nanoparticles (Nut-Mag-SLNs). In particular, we developed a bicompartmental microfluidic device mimicking both the blood vessels and the glioblastoma environment on a single platform. Owning to this device, we were able to assess the ability of the developed Nut-Mag-SLNs to be guided with an external static magnetic field, cross the BBB, reach the glioblastoma compartment, and act as an anticancer agent by reducing cancer cells proliferation while at the same time inducing apoptosis [32].

BoC model can be used to model also non-cancer-related pathologies such as Alzheimer’s disease [33], Parkinson’s disease [10], and brain ischemia [34]. For example, several key hallmarks of Alzheimer’s disease such as the gradual accumulation of amyloid beta protein have been recapitulated in a BoC model based on a side-by-side multichamber microfluidic device [33]. The proposed system was composed of a fluidic chamber seeded with immortalized human brain endothelial cells, two microchannels filled with collagen, and a “brain” compartment with a 3D culture of ReNcell® neuronal progenitor cells expressing Alzheimer’s disease-specific proteins [33]. Similarly, Pediaditakis et al. developed a BoC model to investigate Parkinson’s disease-related alpha-synuclein pathology [10]. The device included a vascular channel seeded with iPSC-derived brain endothelial cells and a brain compartment mimicking the substantia nigra seeded with iPSC-derived dopaminergic neurons, primary human brain astrocytes, microglia, and pericytes. Moreover, the authors were able to reproduce some of the key aspects of synucleinopathies by administrating alpha-synuclein [10]. In the context of neurodegenerative diseases, BoC models can also be used to assess the delivery of candidate compounds and nanoparticles. In one such example, Palma-Florez et al. developed an organ-on-a-chip model for the screening of nanocarriers as theranostic agents against Alzheimer’s disease [35]; the authors developed gold nanorods (GNR) functionalized with polyethylene glycol (PEG), D1 peptide able to inhibit beta-amyloid accumulation, and the BBB targeting angiopep-2 peptide (Ang 2). The BBB crossing ability of the developed nanostructures was assessed on the previously mentioned microfluidic organ-on-chip model composed of brain endothelial cells, astrocytes, and pericytes, and an integrated system for TEER monitoring [35]. In another example, Lenoir et al. developed a Huntington disease (HD) BoC model based on the culture of primary neurons derived from a HD mouse model, and able to replicate the environment of corticostriatal networks [36]. This platform was used to test the efficiency of the drug pridopidine against the synaptic impairments typical of HD. In particular, the authors demonstrated that the administration of pridopidine in their BoC model was able to rescue neurotransmitters trafficking and homeostasis at synaptic level of HD-derived primary neurons [36].

Lyu et al. proposed a BoC model to study stem cell-based regenerative therapies against ischemic stroke. This device was composed of a vascular chamber seeded with immortalized brain endothelial cells, a brain compartment seeded with stem cells-derived neurons, and a third innovative channel mimicking the cerebrospinal fluid (CSF) environment [34]. By exposing the device to low oxygen values and nutrient depletion, the authors were able to simulate some of the hallmarks of ischemic stroke in terms of apoptosis induction gene expression [34].

By using BoC models, the study of more complex cellular behaviors, such as the immune response, is also possible. For example, Lauranzano et al. proposed an NVU model based on a microfluidic bi-compartmental device with a porous filter to study the transmigration of human T lymphocytes across the BBB. The platform is suitable for the evaluation of immune cell trafficking, allowing to test in a brain tumor context whether contact between astrocytes and isogenic T cells during transmigration modulates the molecular and functional phenotype of lymphocytes [37].

Another recent trend in the development of biomimetic *in vitro* microfluidic brain-on-a-chip models is connected to the shift from immortalized cell lines to other cellular sources able to better replicate the physiological characteristics of the human NVU and of the overall CNS. One of the limitations connected with the use of immortalized cell lines is in fact that they are unable to replicate with high fidelity the characteristics of the *in vivo* BBB in terms of permeability and protein expression. Moreover, there have been great efforts from the research community towards the development of personalized *in vitro* BoC models, able to replicate the properties of the NVU and of the surrounding brain parenchyma specific to each patient. Both these points can be addressed by using patient-derived primary cells, like in the case of the aforementioned work by Lauranzano et al., where astrocytes directly isolated from patient brain tissue were co-cultured in a microfluidic model to assess T cell transmigration [37].

Despite their potential, the use of human primary cells is time-consuming due to the complicated and invasive procedure necessary to obtain patient biopsies and the limited proliferative capabilities of primary cells. The advantage of stem cells over primary cells is mainly due to their high proliferative abilities and their capabilities to differentiate into various cell types. For example, iPSCs have been proposed as an alternative to primary cells to set up *in vitro* BBB models. The principle behind the use of iPSCs is to obtain differentiated somatic cells from adults (*e.g*., dermal fibroblast biopsy) to be de-differentiated in iPSCs. iPSCs can then be differentiated again into the various components of the NVU or of the CNS. For example, it has been shown that is possible to reproduce the BBB complex functions using patient-derived iPSCs differentiated into endothelial cells and pericytes, providing a promising platform for studying brain diseases that could potentially be employed to develop personalized therapies [10,11].

Stem cells can be used also to develop self-assembled 3D cultures/co-cultures able to replicate the structure of the brain and NVU. Brain-mimicking models consist of 3D multi-cellular structures obtained from the differentiation of stem cells in NVU components. In particular, two types of human pluripotent stem cells are commonly used to construct organoid-based BBB models: human embryonic stem cells (hESCs) or human iPSCs (hiPSCs), which are then differentiated into the various components of the NVU or in other cellular components of the CNS, such as specific subsets of neurons. The most recent trends involve the vascularization of the models to better replicate the functional NVU *in vitro*. The main strategies involve the use of growth factors to induce vessel-like structures [38]; alternatively, more recently it has been demonstrated that vascularization can be induced by co-culturing fragments of vascularized organoids with no-vascularized organoids [39]. Being easy to set up and relatively economical, 3D brain-mimicking models can also be used in high-throughput screening approaches where a vast array of candidate drugs and substances can be simultaneously assessed [40]. For example, in 2018 Bergmann et al. described a protocol to produce BBB organoids to test the brain permeability of candidate compounds [41]. The protocol described in this work was based on the co-culture of brain endothelial cells, astrocytes, and pericytes under low-adhesion conditions. The obtained organoids were able to express key molecular factors involved in the transport mechanisms across the BBB, and could be used for the high-throughput screening of drug permeability through fluorescence-based techniques or mass spectrometry imaging [41].

Although the discussed models have provided valuable insights, a major limitation is their large size compared to brain capillaries. To overcome this issue different strategies to obtain BoC models with sizes comparable to those of brain capillaries have been proposed. For example, self-assembled capillaries have been developed by Campisi et al. [42]. These capillaries are composed of a monolayer of hiPSC-derived ECs, co-cultured with brain PCs and ACs embedded in a 3D fibrin hydrogel. The resulting BBB model exhibits a selective microvasculature able to be perfused with permeability levels lower than conventional *in vitro* models, and comparable to *in vivo* measurements. Furthermore, the 3D microfluidic system has been exploited to measure the passage of differently-sized nanoparticles across the BBB [43].

Lastly, our group successfully demonstrated the potential of high-resolution 3D printing technologies in creating biomimetic fluidic brain microcapillaries [44]. We exploited two-photon lithography to fabricate structures at the submicron scale, and developed the first 1:1 scale biomimetic BBB. The proposed microfluidic device is composed of multiple parallel porous microcapillaries, where HBMECs were seeded on the inner surface. Recently, the BBB model has been further enhanced by incorporating 3D magnetic scaffolds to enable tumor cell culture and to mimic the interaction between BBB and glioblastoma [45]. The developed BoC model was used to test the permeability and therapeutical efficiency of anticancer drugs against GBM.

Details of the discussed microfluidic platforms, such as the type of cells used, the parameters analyzed to evaluate barrier integrity, and applications are listed in Table 1. The main future perspective in the development of BBB biomimetic *in vitro* models is represented by the combination of the approaches described in this section. In particular, the future ideal *in vitro* BBB model should include several parallel BBB platforms for high-throughput screening, where each platform should be composed of biomimetic, tridimensional, and multicellular models in 1:1 scale with brain capillaries.

## 4 Integration of sensors in BoC models

**Fig. 3:**
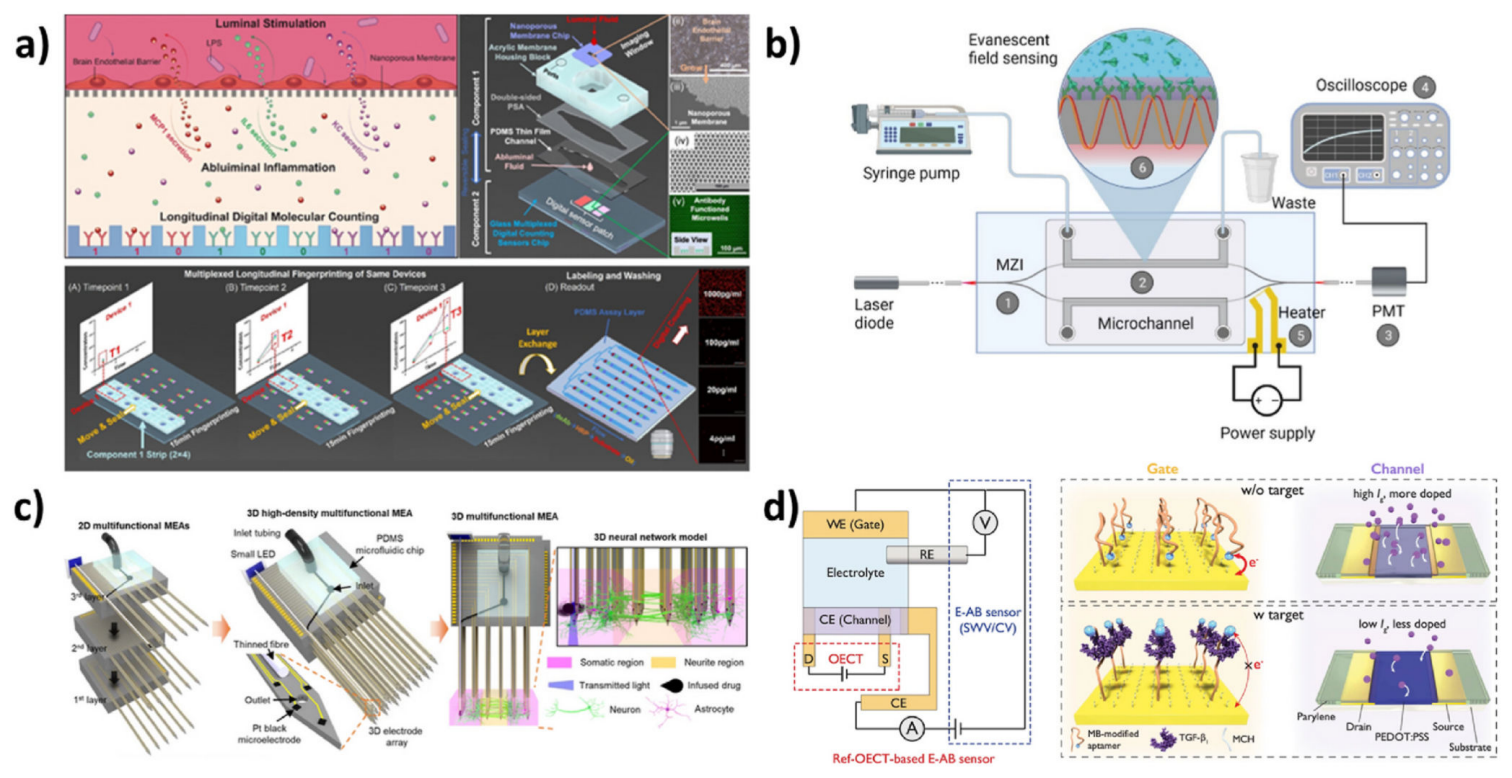
Representative sensors designed for integration into BoC models. a) Schematic of digital immunosensors integrated into BBB-on-a-chip model for the sequential detection of cytokines. Reprinted with permission from. Ref. [69]. b) Schematic representation of an integrated optical Mach-Zender interferometer for the detection of target spike protein S1 subunit crossing the BBB after coronavirus infection. Reprinted with permission from Ref. [70]. c) Schematic illustration of 3D high-density microelectrode array integrated into a 3D neural network *in vitro* model. Reprinted with permission from Ref. [71]. d) Schematic of the sensing mechanisms of the ref-OECT-based E-AB sensor for the detection of TGF-B1. Reprinted with permission from Ref. [52].

Recent advances in sensors have been of great interest in the study of disease progression in response to drug treatments. Their integration into microfluidic BoC models enables real-time monitoring of tissues (Fig. 3), while previous approaches were mainly limited to end-point tests, reducing the gap between in vitro and in vivo models. Sensors aim to be highly sensitive, accurate, stable over time, and minimally invasive. They are classified into three categories depending on their detection method: electrical, electrochemical, or optical [46].

Electrical sensors have been extensively used in BoC models due to their simple integration into microfluidic models. Microelectrode arrays (MEA) are particularly well adapted to measure the electrophysiological activity of neurons, but can also provide information on BBB integrity [8,46]. Recent studies showed the potential of such models in the scope of brain diseases. Liu et al. recently combined indium-tin-oxide microelectrodes with neural stem cells spheroid-based BoC models to study AD. Real-time impedance measurements were correlated with the formation and degeneration of neuronal networks after the addition of amyloid beta, one of the major pathological hallmarks of AD known to be toxic for neurons and to induce degeneration of synapses [47]. Still regarding AD treatment, Palma-Florez et al. integrated microelectrodes into their BBB-on-a-chip to evaluate the permeability performance of gold nanorods developed to overcome the BBB and to disassemble amyloid aggregates. The measure of TEER with electrodes placed at a micrometric distance of the BBB allowed them to prove the successful formation of a cell barrier and to evaluate the effect of nanomaterials on the tight junctions’ integrity [48].

Electrochemical biosensors are a sub-class of electrical sensors where the working electrode is modified with a biological recognition element [49]. A recent trend is represented by the implementation of miniaturized aptamer-beacon-based (E-AB) sensors for high-sensitive screening purposes. These rely on modified electrodes functionalized with aptamers, which are synthetic DNA or RNA strains able to bind target molecules with high affinity and selectivity. The binding of specific target molecules with redox-labeled aptamers induces a change in the 3D conformation, modifying the distance between the redox tag and the electrodes and thus the measured signal. These types of sensors have been widely used, as they offer real-time sensing of a broad range of targets. However, their sensitivity is highly dependent on the number of aptamers and therefore on the surface area, limiting the possibility of miniaturization for the integration into BoC devices. To overcome this limitation, Guo et al. developed a 2D CuNi metal-organic framework exhibiting a graphene-like structure, able to anchor a much larger amount of aptamers compared to standard electrodes of similar size. With this bifunctional sensor, they selectively detected living C6 glioma cells (from 50 to 1.105 cells/mL) and their endothelial growth factor (EGFR) biomarker (limit of detection of 0.72 fg/mL), exhibiting great potential for cancer diagnosis [50]. In 2022, Shaver et al. optimized an aptamer-based sensor to reach a better detection of vancomycin, an antibiotic that can be used for the treatment of bacterial meningitis. By using a one-base-pair-longer aptamer, they were able to resolve vancomycin concentration from baseline noise in the brain cortex of living mice, which was not possible in previously developed vancomycin-E-ABs [51]. Finally, most recently, Ji et al. used organic electrochemical transistors as on-site amplifiers for the detection of transforming growth factor beta 1 (TGF-β1). Their device consists of an aptamer-modified Au working electrode, an Ag/AgCl reference electrode, and a poly(3,4-ethylenedioxythiophene)-poly(styrenesulfonate) (PEDOT: PSS) counter-electrode, achieving direct amplification of the current in the working electrode [52]. They could reach 3-4 orders of magnitude enhancement in sensitivity compared to the bare E-AB sensor. These different approaches pave the way for future integration of E-AB into BoC, and can also be applied to other types of electrochemical sensors to improve electrode interface and implement in situ amplification of measured signals.

Electrochemical sensors can be also designed to detect small metabolic products, such as reactive oxygen species (ROS) and reactive nitrogen species (RNS), which regulate a plethora of biological phenomena at physiologic concentrations (e.g., H2O2 intracellular concentration in the 1–100 nM range), yet contribute to the pathogenesis of different neurodegenerative diseases at elevated concentrations [53,54]. The overproduction of these reactive molecules triggers oxidative damage to different cell components, such as DNA bases, amino acids, and phospholipids of the membranes, with activation of senescence and apoptotic pathways. Due to the high reactivity of these molecules, their half-life in the cell medium is very short: therefore, sensors require elevated sensitivity [55]. In this regard, electrochemical sensors allow the local detection of ROS/NOS in real-time with high selectivity, elevated sensitivity, and miniaturization potential [56]. The development of these sensors recently reached the single-cell detection scale thanks to the use of nanoelectrodes [57]. The most investigated ROS/NOS with this technology is H2O2, which is also the most common and stable ROS. Two types of H2O2 electrochemical sensors have been developed: the enzymatic biosensors, such as those based on the horseradish peroxidase, show good sensitivity but limited stability and potential inhibition of the enzymatic activity by contaminants [55]; the non-enzymatic sensors, instead, show long-term stability. Furthermore, different materials/nanomaterials-composites have been recently exploited in non-enzymatic H2O2 sensors to reach excellent sensitivity properties [58–65]. Concerning CNS application, despite different H2O2 sensors have been exploited to monitor the ROS levels in brain cancer cells (such as U87 glioblastoma cells), the incorporation of these sensors in proper 3D brain tumor-a-chip (BToC) or BoC models has not been described yet. However, since the ROS are known to play a fundamental role in the regulation of neuronal development and function (i.e., cell polarity, axonal outgrowth, synapsis formation, and network tuning), the approach of monitoring their concentration in real-time by electrochemical sensors is expected to be increasingly used due to their improved performances; also the rapid commercialization and standardization of such systems for high-throughput tests is envisaged.

Optical sensors are easily integrable into BoC models as they do not require direct physical or electrical contact between the transducer and the detector [8,66]. The optical signal is generated directly by the interaction of the analyte with the transducer or through a labeling agent. Recent progress in gene engineering led to the development of genetically encoded fluorescent indicators with a high spatiotemporal resolution, providing access to a broad range of biological events. Such indicators have been recently used for calcium imaging, detection of neural activation (voltage-sensitive indicators), and as sensors for neurotransmitters and neuromodulators with a high signal-to-noise ratio on in vitro and in vivo brain models [67]. Most recent trends concern the expansion of the spectral diversity, through the development of a genetically-encoded far-red fluorescent indicator for the imaging of synaptic Zn^2+^, a key neuromodulator in the brain [51], or a near-infrared genetically-encoded indicator for calcium imaging [68]. An innovative optical biosensor was recently presented by Su et al. in 2023. They developed a BBB-on-a-chip platform integrating digital immunosensors for the sequential detection of three cytokines species relevant to neuroinflammation, with a limit of detection of 100-500 fg/mL. These immunosensors have a short detection time and use a very limited amount of bulk solution to generate digital sensor signals [69].

Mach-Zehnder interferometer is also of great interest regarding its high sensitivity and portability, facilitating its integration into BoC models. This interferometric construction is based on waveguide structures functionalized by recognition elements. The binding of analytes induces a change of the refractive index in the measuring arm, resulting in a phase shift in the propagation wave compared to the referring arms. This type of device was recently integrated into a BBB-on-a-chip model to demonstrate the barrier penetration capability of the surface spike protein subunit 1 of SARS-CoV-2 into the brain, emphasizing the neuroinvasive potential of this virus [70].

## 5 Discussion

Biomimetic BoC and BBB-on-a-chip are platforms allowing researchers for extensive investigations and predictive experiments, such as testing brain delivery and efficacy of drugs/nanoformulations, reconstructing disease conditions, evaluating immunotherapy responses, mimicking tumor-induced angiogenesis, and studying interactions among tumor and healthy cells. Recent trends include i) biomimicry to improve prediction capabilities (e.g., by incorporating hiPSCs, patient-derived cells, 3D organoids, and tissue-specific ECM matrix components), and ii) sensorization for real-time monitoring and/or high-throughput screening of molecule release (e.g., nanomodulators, nanotransmitters, growth factors, cytokines), receptor expression, cell viability, cell-cell interactions, intracellular phenomena (e.g., calcium waves) and BBB crossing of drugs, nanoparticles, and viruses.

**Fig. 4:**
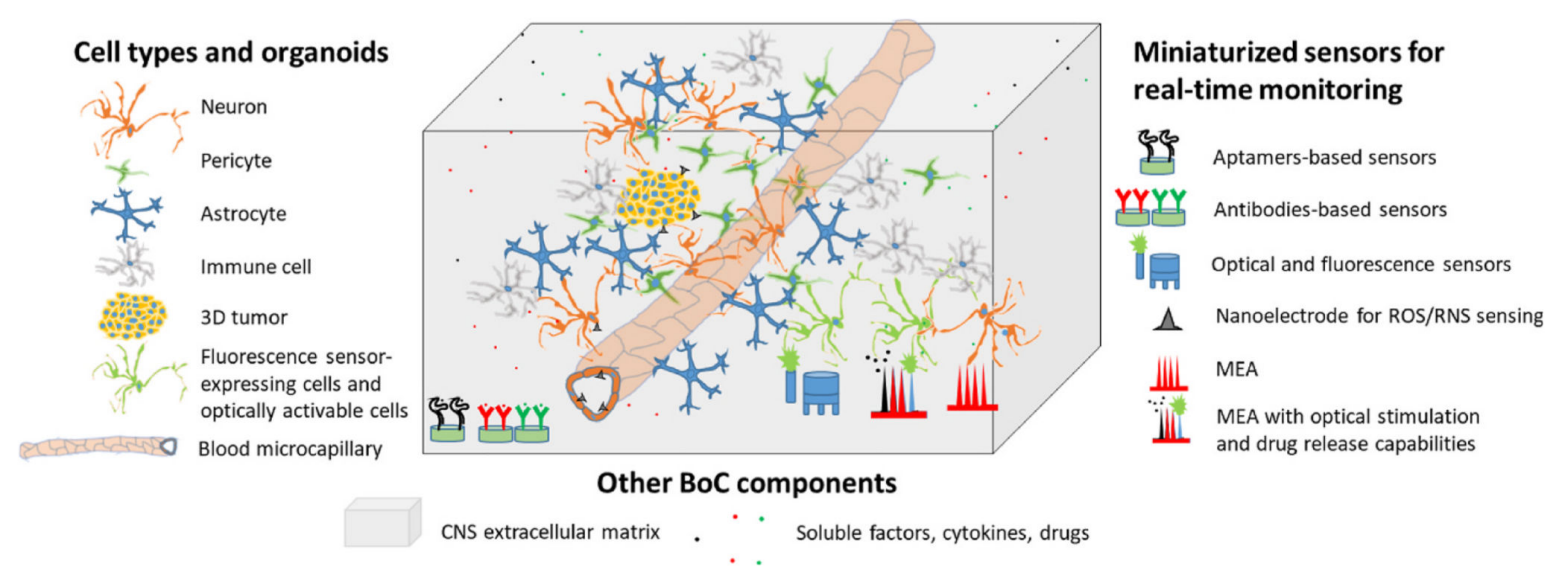
Schematic representation of different sensors integrated into a biomimetic BoC.

Regarding sensorization, electrical, electrochemical, and optical sensors have benefits and limitations that must be considered for their integration into BoC devices. In Fig. 4, a schematic representation showing integration of different sensors into different compartments of biomimetic BoC has been provided. Electrical sensors are the easiest to integrate but they provide non-specific information. Miniaturization of the electrodes improves spatial resolution but induces high impedance and thus a decreased recording quality. Recent approaches improved the sensitivity by in situ amplifying the produced signal or by using porous conductive materials that increase the surface area and lower the impedance. Implementation of 3D MEAs is also considered for better spatial coverage of 3D models, and the use of thin-film electrodes that are less invasive is very promising for better integration into BoC models [71]. Electrical sensors measuring neural activity are appropriate for the multi-compartmental systems, where the pumped fluid is maintained separated and does not interfere with the working electrode. Their application is particularly appealing for the BoC models developed from human stem cells to monitor their electrophysiologic maturation into neurons. Furthermore, they can be also used with matured neurons to detect their functionality after drug treatment.

Electrochemical sensors exhibit similar characteristics, as they rely on the same principle. However, they require an additional functionalization of the electrodes with specific biological recognition elements, on which the measurement is greatly dependent. Recent studies investigated different approaches to improve the functionalization, allowing miniaturization of the sensors. Electrochemical sensors may be integrated in the presented BoC models in different configurations: i) biosensors can be positioned in the “brain” mimicking compartment for real-time molecule/drug detection after BBB crossing; ii) miniaturized electrochemical sensors, such as in the form of nanoelectrodes, can be implemented in the “blood” compartment to locally detect ROS levels in endothelial cells (e.g., for analyzing the safety of the delivered drug at microcapillary level of the BBB). Another envisaged application of miniaturized electrochemical sensors is the real-time monitoring of the neurodegeneration in pathologic models, such as those mimicking Alzheimer’s and Parkinson’s diseases, and the detection of potential functional rescue with specific treatments. Similarly, the detection of ROS and RNS with miniaturized sensors can be applied to brain tumor-on-a-chip models to monitor 3D tumor response to anticancer drugs.

Optical biosensors are probably the less commonly integrated into BoC models, as they require much more complex techniques and optical read-out systems. However, they are very promising as they are less invasive, they consume few or no analytes, and the sensing area can be miniaturized to a few micrometers. Combining several of the recently developed biosensors into a single microfluidic platform seems to be very promising for improving the real-time monitoring of BoC models in response to drug treatments, and should be the challenge of these next years. Finally, multimodal stimulation and sensing systems have been recently developed for the monitoring of neural circuit responses within engineered 3D neural tissues. An example is the use of 3D MEAs integrated with a small light-emitting diode (LED) for optogenetic neuromodulation and microfluidic channels with pressure-driven drug delivery capabilities [71]. This approach allows for precise mapping of functional connectivity in the entire 3D tissue volume with improved recording performances thanks to the Pt black covering of the electrodes. Such integrated stimulation recording systems represent a valuable tool to study the response of neural tissue functionality to different drugs, and can be potentially applied to both physiologic and pathologic BoC models without the need for further microfluidic integration. The main limit of this delivery system is that it cannot provide direct information on BBB permeability capabilities and drug functionality after BBB crossing; however, this MEA coupled with a delivery system can be potentially downstream connected to a BBB screening device able to provide such functions.

## 6 Conclusions

BoC and BBB-on-a-chip models will support more and more researchers in the pre-clinical testing of novel therapies for CNS. Thanks to the incorporation of hiPSCs and patient-derived cells in these devices, new patient-tailored approaches can be discovered and tested. Also, signal amplification, miniaturization, and multiplication of the sensors in BoC are the key processes to increase the types of biological phenomena that can be simultaneously detected. The standardization of BoC tests may also simplify and improve the result replicability in different laboratories, which can be complex to obtain in the case of customized bioreactors. Finally, the implementation of miniaturized optical sensors and their combination with genetically-encoded fluorescent indicators, despite being technically challenging, is expected to be the next frontier of BoC, since it would allow monitoring of a plethora of complex biologic phenomena and intracellular pathways.

## Figures and Tables

**Table 1:** Summary of the main characteristics of the microfluidic brain-on-a-chip models.

Ref	Trend	Aim	Configuration of the BBB	Fabrication/set-up procedure	3D ECM material	Cellular NVU components	Other cell types
[29]	3D microfluidic device with human cells	Establishment of an NVU *in vitro* model and test of nanoparticle passage	Double-layer	Photolithography/soft-lithography (PDMS)	Matrigel	Immortalized: HBMECs Primary: HBVPs, HAs	N/A
[30]	3D microfluidic device with human cells	Set-up of *an in vitro* BBB model able to mimic CNS angiogenesis	Side-by-side	Photolithography/soft-lithography (PDMS)	Fibrin hydrogel	Primary: HBMECs, HUVECs, human pericytes, HAs	3D fibroblast spheroids as a source of angiogenic factors
[31]	3D microfluidic device with human cells, incorporation of a brain compartment mimicking GBM	Study of the interactions between GBM and NVU, and study of drug permeability	Tubular	Photolithography/soft-lithography (PDMS)	Collagen type I	Primary: HBVPs, HAs, HBMECs	Spheroids of T98G and U87MG cells
[32]	3D microfluidic device incorporating a brain compartment mimicking GBM	Drug delivery tests involving lipid nanostructures for the treatment of glioblastoma	Double-layer	Laser cut and assembly of a multichamber device based on a porous insert	N/A	Immortalized: bEnd.3 mouse brain endothelial cells	U87MG cells
[33]	3D microfluidic device with human cells, incorporation of a brain compartment mimicking AD	Set-up of an *in vitro* model of AD	Side-by-side	Photolithography/ soft-lithography (PDMS)	Collagen type I	Immortalized: hCMEC/D3	ReNcell® human neural progenitor cell line either wild-type or AD-related mutations
[10]	Microfluidic device with human cells, incorporation of a brain compartment mimicking AD, use of stem cells	Set-up of an *in vitro* model of PD	Double-layer	Fabricated by Emulate, Inc. (PDMS)	N/A	hiPSC-derived HBMECs Primary: HAs, HBVPs, human microglia	iPSCs-derived dopaminergic neurons
[34]	3D microfluidic device with human cells, incorporation of a brain compartment, use of stem cells	Establishment of an ischemia model and testing of a stem cell-based regenerative therapy	Side-by-side	Stereolithography 3D printing (PDMS)	Hydrogel	Primary: BMECs, Has, human brain vascular pericytes	HMC3, stem cells-derived neurons
[35]	3D microfluidic device with human cells	Development of a microfluidic NVU model for testing drug delivery platforms	Side-by-side	Photolithography/soft lithography (PDMS)	Hydrogel	Immortalized: hCMEC/D3 Primary: human hippocampal astrocytes, human brain-vascular pericytes	N/A
[36]	3D microfluidic device with human cells	Development of a HD BoC model for drug delivery tests	Side-by-side	Photolithography/soft lithography (PDMS)	N/A	N/A	cortical and striatal neurons
[37]	3D Microfluidic device with human cells, incorporation of immune cells	NVU with patient- derived astrocytes obtained from tumor resection margin. Model used to test T lymphocyte migration	Device-based on porous membrane insert exposed to flow conditions	Assembly of a multichamber device based on a porous insert	Geltrex	Immortalized: hCMEC/D3 Primary: HAs	Peripheral blood mononuclear cells (PBMCs)
[11]	Microfluidic device with human cells, incorporation of a brain compartment	Establishment of a personalized BoC model as a potential platform for disease modeling and drug screening	Double-layer	Fabricated by Emulate, Inc. (PDMS)	N/A	hiPSC-derived HBMECs Primary: HAs, human pericytes	iPSC-derived neural cells
[38]	3D vascularized organoids based on human cells, use of stem cells	Set-up of vascularized human cortical organoids	Organoids based on hESCs	Engineered hESCs	N/A	hESCs-derived ECs	hESCs differentiated into cells expressing markers typical of cells of the brain environment like cortical neurons, interneurons, astrocytes, radial glial cells, glial progenitor cells, and neuronal progenitor cells
[39]	3D vascularized organoids based on human cells, use of stem cells	Analysis of the interaction between BVOs and cortical organoids	Organoids based on hiPSCs	Differentiation of hiPSCs	Matrigel	hiPSCs-derived blood vessel organoids (BVOs)	hiPSCs-derived cortical organoids
[40]	3D organoids, use of human cells	Set-up of BBB organoids for drug screening	Organoids based on differentiated cells	Assembly of the cellular components of the NVU into organoids	Hydrogel	Immortalized: hCMEC/D3 Primary: human pericytes,HAs	N/A
[41]	3D organoids, use of primary human cells	Set-up of BBB organoids for drug screening	Organoids based on the selfassembly properties of NVU components	Self-assembly of the cellular components of the NVU into organoids	3D structures based on the self-assembly properties of NVU cells	Immortalized: hCMEC/D3 Human brain vascular pericytes (HBVPs), Primary human astrocytes	N/A
[43]	1:1 scale, 3D microfluidic device with human cells, use of stem cells.	Set-up of an NVU microfluidic model for testing the passage of nanostructures	Side-by-side	Photolithography/ soft-lithography (PDMS)	Fibrin hydrogel	hiPSC-HBMECs Primary: HAs, human pericytes	N/A
[45]	1:1 scale, 3D microfluidic device with human cells, incorporation of a brain compartment	Set-up of a brain-on-a- chip model in 1:1 scale with brain capillaries for drug-screening	Tubular	Two-photon lithography (SU-8 photoresist)	N/A	Immortalized: hCMEC/D3 Primary: HAs	U87MG glioblastoma cells

## Data Availability

No data was used for the research described in the article.

## Abbreviations

eHNP-A1: Apolipoprotein A1
E-AB: aptamer-beacon-based
BBB: blood-brain barrier
BoC: brain-on-a-chip
CNS: central nervous system
CSF: cerebrospinal fluid
EGFR: epidermal growth factor receptor
GBM: glioblastoma multiforme
HAs: human astrocytes
HBMECs: human brain microvascular endothelial cells
HBVPs: human brain vascular pericyte
hESCs: human embryonic stem cells
iPSCs: induced pluripotent stem cells
NVU: neurovascular unit
PEDOT: PSS: poly(3,4-ethylenedioxythiophene)-poly (styrenesulfonate)
TEER: transendothelial electrical resistance
TGF-β1: transforming growth factor β 1
